# Detection of Circulating B Cells Producing Anti-GPIb Autoantibodies in Patients with Immune Thrombocytopenia

**DOI:** 10.1371/journal.pone.0086943

**Published:** 2014-01-22

**Authors:** Masataka Kuwana, Yuka Okazaki, Yasuo Ikeda

**Affiliations:** 1 Division of Rheumatology, Department of Internal Medicine, Keio University School of Medicine, Tokyo, Japan; 2 Faculty of Science and Engineering, Waseda University, Japan; Pavillon Kirmisson, France

## Abstract

**Background:**

We previously reported that an enzyme-linked immunospot (ELISPOT) assay for detecting anti-GPIIb/IIIa antibody-secreting B cells is a sensitive method for identifying patients with immune thrombocytopenia (ITP). Here we assessed the clinical significance of measuring circulating B cells producing antibodies to GPIb, another major platelet autoantigen.

**Methods:**

Anti-GPIb and anti-GPIIb/IIIa antibody-producing B cells were simultaneously measured using ELISPOT assays in 32 healthy controls and 226 consecutive thrombocytopenic patients, including 114 with primary ITP, 25 with systemic lupus erythematosus (SLE), 30 with liver cirrhosis, 39 with post-hematopoietic stem cell transplantation (post-HSCT), and 18 non-ITP controls (aplastic anemia and myelodysplastic syndrome).

**Results:**

There were significantly more circulating anti-GPIb and anti-GPIIb/IIIa antibody-producing B cells in primary ITP, SLE, liver cirrhosis, and post-HSCT patients than in healthy controls (*P*<0.05 for all comparisons). For diagnosing primary ITP, the anti-GPIb ELISPOT assay had 43% sensitivity and 89% specificity, whereas the anti-GPIIb/IIIa ELISPOT assay had 86% sensitivity and 83% specificity. When two tests were combined, the sensitivity was slightly improved to 90% without a reduction in specificity. In primary ITP patients, the anti-GPIb antibody response was associated with a low platelet count, lack of *Helicobacter pylori* infection, positive anti-nuclear antibody, and poor therapeutic response to intravenous immunoglobulin.

**Conclusion:**

The ELISPOT assay for detecting anti-GPIb antibody-secreting B cells is useful for identifying patients with ITP, but its utility for diagnosing ITP is inferior to the anti-GPIIb/IIIa ELISPOT assay. Nevertheless, detection of the anti-GPIb antibody response is useful for subtyping patients with primary ITP.

## Introduction

Immune thrombocytopenia (ITP) is an autoimmune disease in which accelerated platelet consumption and impaired platelet production are mediated primarily by IgG anti-platelet autoantibodies [1]. This condition is seen in patients with various diseases, including systemic lupus erythematosus (SLE) and human immunodeficiency virus infection. It can also occur without an underlying disease, which is known as primary ITP. The production of IgG autoantibodies to platelet surface glycoproteins, such as GPIIb/IIIa and GPIb, is the hallmark of the disease [2]. Several antigen-specific assays for detecting platelet-associated anti-GPIIb/IIIa and anti-GPIb antibodies are reported to be useful for identifying patients with ITP [3]–[5]. However, no laboratory test for detecting platelet antigen-specific antibodies is used widely in clinical settings, because these assays require complicated procedures such as platelet solubilization, the use of commercially unavailable monoclonal antibodies, and a relatively large blood sample.

We previously developed an enzyme-linked immunospot (ELISPOT) assay for detecting IgG anti-GPIIb/IIIa antibody-secreting B cells in the circulation and spleen of patients with primary ITP [6]. We subsequently showed that the detection of circulating anti-GPIIb/IIIa antibody-producing B cells is a sensitive, specific, and convenient method for evaluating the presence or absence of an anti-platelet autoantibody response [7]. The anti-GPIIb/IIIa antibody response is very common in patients with primary ITP as well as those with various forms of secondary ITP, including thrombocytopenia associated with SLE [8], liver cirrhosis with or without hypersplenism [9], and post-hematopoietic stem-cell transplantation (post-HSCT) [10]. These findings led us to propose preliminary diagnostic criteria for ITP based on a combination of ITP-associated laboratory findings, including circulating anti-GPIIb/IIIa antibody-producing B cells, reticulated platelets, and thrombopoietin [11]. The ELISPOT assay has several advantages over assays that detect platelet antigen-specific antibodies, i.e., the results are not influenced by the binding of the antibodies to platelet surfaces and only a small blood sample (<3 mL) is required. However, the anti-GPIIb/IIIa antibody response was not detectable in a small proportion (∼20%) of ITP patients, even if the sensitive ELISPOT assay was used. Thus, adding a concomitant measurement of B cells producing antibodies to another major platelet autoantigen, GPIb, may increase the assay’s sensitivity to the anti-platelet autoantibody response in patients with ITP. In this study, we established an ELISPOT assay for detecting anti-GPIb antibody-producing B cells and evaluated its potential usefulness for the diagnosis, disease subtyping, and assessment of the anti-platelet autoantibody profiles in patients with primary ITP and a various forms of secondary ITP.

## Materials and Methods

### Subjects

This study included 114 consecutive patients with primary ITP whose peripheral blood samples had been sent to an autoimmune laboratory at Keio University Hospital between April 2003 and March 2005. Eighteen patients were also included in a multicenter prospective study for verification of our preliminary diagnostic criteria for ITP [11]. The inclusion criteria were: (i) clinical diagnosis of primary ITP; (ii) thrombocytopenia (platelet count ≤50×10^9^/L); (iii) no previous treatment with corticosteroids or immunosuppressants; and (iv) availability of detailed clinical information for at least one year after the diagnosis. The clinical diagnosis of ITP was made by one of the authors (YI) on the basis of clinical history, physical examination, complete blood test, and bone marrow findings if available, according to the guidelines proposed by the American Society of Hematology [12]. The final diagnosis was re-evaluated, taking into account the clinical course of the disease over at least one year, especially the therapeutic responses to corticosteroids, splenectomy, and eradication of *Helicobacter pylori* (*H. pylori*). YI was blinded to the results of the anti-GPIIb/IIIa and anti-GPIb antibody-producing B cell assays, so the clinical diagnosis of primary ITP was not influenced by these laboratory findings. Patients with primary ITP were classified as having newly diagnosed, persistent, or chronic ITP, as described previously [13].

Additional thrombocytopenic patients with underlying diseases that could potentially cause secondary ITP or non-ITP thrombocytopenia were selected from consecutive patients whose peripheral blood samples had been sent to the autoimmune laboratory during the same period, based on the definitive diagnosis of underlying diseases/conditions and platelet count ≤50×10^9^/L. SLE and liver cirrhosis were diagnosed according to the published criteria [14], [15]. HSCT recipients were selected based on a lack of sustained anemia or leukopenia, and no apparent cause for thrombocytopenia, such as engraftment failure, recurrence of the underlying hematologic malignancy, microangiopathy, or drugs [10]. To minimize the potential influence of procedure-related complications, we selected patients who had survived for >100 days after HSCT. Patients with aplastic anemia or myelodysplastic syndrome (MDS) were also enrolled as a non-ITP disease control. Diagnosis of aplastic anemia and MDS was based principally on bone marrow findings and cytogenetic analysis [16], [17]. Thirty-two healthy individuals were also included as a control. All samples were obtained after the subjects gave their written informed consent, as approved by the ethical committee of Keio University School of Medicine (Application number 2010-031-2).

### Therapeutic Response

A therapeutic response to intravenous immunoglobulin (IVIG) was defined as a platelet count >100×10^9^/L at one week, respectively [8], while a response to *H. pylori* eradication or corticosteroids (>0.5 mg/kg prednisolone in combination with or without IVIG) was defined as a platelet count >100×10^9^/L at 24 weeks, respectively [18]. We used the published definition for therapeutic response to splenectomy [19], but complete and partial responses were combined. That is, a therapeutic response was defined as a platelet count of ≥50×10^9^/L for 30 days or longer after splenectomy, with or without other treatment.

### *H. pylori* Infection

*H. pylori* infection was evaluated with a ^13^C urea breath test using a UBiT tablet (Otsuka Assay, Tokyo, Japan), the detection of serum IgG anti-*H. pylori* antibodies using a commercially available kit (Kyowa Medex Company, Tokyo, Japan), and the detection of *H. pylori* antigen in stool samples using ImmunoCard® HpST® (Meridian Bioscience, Cincinnati, OH). Patients positive for the urea breath test plus at least one additional test were regarded as *H. pylori*-positive [18].

### Antinuclear Antibody (ANA)

ANA was measured by indirect immunofluorescence using commercially available HEp-2 slides (MBL, Nagoya, Japan) as the substrate. A positive result was determined as a significant signal using two different cut-off levels: serum samples diluted 1∶40 and 1∶160.

### Measurement of IgG Anti-GPIIb/IIIa and Anti-GPIb Antibody-producing B Cells

B cells producing IgG anti-GPIIb/IIIa antibodies were measured using the ELISPOT assay as described previously [6], [7]. Briefly, a polyvinylidene difluoride-bottomed 96-well microplate (Millipore, Bedford, MA) was activated by incubation with ethanol (>99.5%) at room temperature for 10 minutes. After extensive wash with phosphate-buffered saline (PBS) containing 0.5 mM CaCl_2_ (PBS-Ca), the microplates were coated with affinity-purified human GPIIb/IIIa (purity >80%; Enzyme Research Laboratories, Swansea, UK) dissolved in PBS-Ca at a concentration of 30 µg/mL over night at 4°C. Then, the plates were washed three times with PBS-Ca, and were subsequently blocked with 1% bovine serum albumin (Sigma-Aldrich, St. Louis, MO) in PBS-Ca at room temperature for one hour. Peripheral blood mononuclear cells (PBMCs), isolated from heparinized peripheral blood by Lymphoprep (Fresenius Kabi Norge AS, Halden, Norway) density gradient centrifugation, were re-suspended in RPMI1640 containing 10% fetal bovine serum (Life Technologies, Carlsbad, CA), and were pipetted into the wells (10^5^/well) and cultured at 37°C with 5% CO_2_ for 4 hours. After washing away the cells with PBS-Ca containing 0.05% Tween 20, the membranes were incubated with alkaline phosphatase-conjugated goat anti-human IgG (ICN/Cappel, Aurora, OH) diluted at 1∶1,000 in PBS-Ca at room temperature for 2 hours, followed by wash two time each with PBS-Ca with 0.05% Tween 20 and PBS-Ca. Finally, anti-GPIIb/IIIa antibodies that bound to the membrane were visualized as spots by incubation with nitro blue tetrazolium (Sigma-Aldrich; 300 µg/mL)/5-bromo-4-chloro-3-indolyl phosphate (Sigma-Aldrich; 60 µg/mL) in a buffer consisting of 100 mM Tris-HCl (pH9.5), 100 mM NaCl, 50 mM MgCl_2_ at room temperature for 20 minuts. B cells producing IgG anti-GPIb antibodies were also measured by ELISPOT assay, in which a recombinant GPIbα fragment was used instead of GPIIb/IIIa as the antigen. The recombinant GPIbα fragment, which covered the entire von Willebrand factor-binding site (residues 1 to 302), was expressed in Chinese hamster ovary cells [20]. For the anti-GPIb antibody ELISPOT assay, PBS was used instead of PBS-Ca in the entire protocol. The plates coated with bovine serum albumin in the blocking buffer in the absence of GPIIb/IIIa or GPIb were used as control for the ELISPOT assay. Each assay was conducted in 5 independent wells, and the results represented the mean of the 5 values. The frequency of circulating anti-GPIIb/IIIa or anti-GPIb antibody-producing B cells was calculated as the number per 10^5^ PBMCs. The cut-off value for anti-GPIIb/IIIa antibody-producing cells was defined as 2.0 per 10^5^ PBMCs [7]. The cut-off value for anti-GPIb antibody-producing cells was set at 5 standard deviations above the mean value from healthy controls.

### Statistical Analysis

All continuous variables were expressed as the mean ± standard deviation (SD). Comparisons between two groups were tested for statistical significance using the Mann-Whitney test. Differences in frequency between two groups were compared using the Chi-square test or Fisher’s exact test, when applicable. The correlation coefficient (r) was determined using a single-regression model.

## Results

### Patient Characteristics

This study enrolled a total of 226 thrombocytopenic patients. They were composed of 114 with primary ITP, 25 with SLE, 30 with liver cirrhosis, 39 with post-HSCT, and 18 non-ITP controls, including 4 with aplastic anemia and 14 with myelodysplastic syndrome. Table 1 summarizes the sex, age at examination, and platelet count of thrombocytopenia patients and healthy controls. Forty-eight patients (42%) with primary ITP were categorized as having newly diagnosed ITP, while the remaining patients had persistent or chronic ITP. The etiologies of liver cirrhosis included hepatitis B virus infection in 5, hepatitis C virus infection in 21, and alcohol in 4. Of the post-HSCT patients, 37 had received bone marrow transplantation while 2 had received peripheral blood stem cell transplantation. Compared with patients with primary ITP, patients with SLE were predominantly female (*P* = 0.02) and those with liver cirrhosis and MDS were older (*P* = 0.001 and *P* = 0.03, respectively). There was no difference in platelet count among the thrombocytopenic patient groups. The mean follow-up period in patients with primary ITP was 49±26 months.

**Table 1:** Demographic features and platelet count of subjects enrolled in this study.

	Number	Sex (% male)	Age at examination (years)	Platelet count (×10^9^/L)
Primary ITP	114	40%	49.6±17.1	28.1±11.6
SLE	25	12%	43.6±14.6	28.6±13.3
Liver cirrhosis	30	53%	63.3±9.1	35.2±11.5
Post-HSCT	39	59%	37.6±10.6	31.5±11.3
Aplastic anemia	4	25%	46.3±23.9	24.5±16.2
MDS	14	57%	60.4±17.5	26.9±13.3
Healthy controls	32	50%	44.1±12.2	252.4±56.8*

ND: not determined.

* Data were derived from 16 healthy donors.

### Detection of IgG Anti-GPIIb/IIIa and Anti-GPIb Antibody-producing B Cells

**Figure 1:**
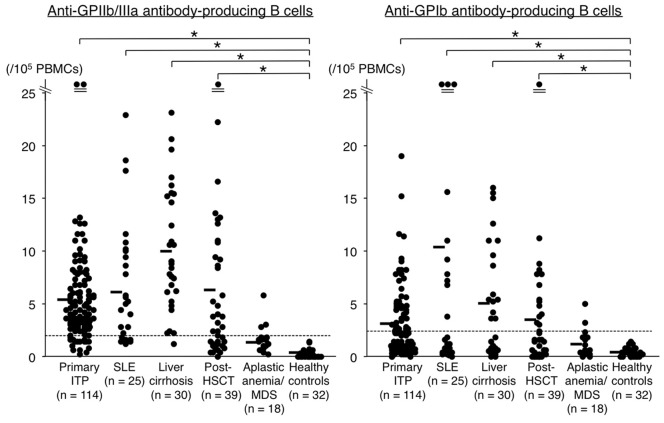
Anti-GPIIb/IIIa and anti-GPIb antibody-producing B cells in the circulation of patients with various thrombocytopenic conditions and healthy controls. Cut-off values for anti-GPIIb/IIIa and anti-GPIb antibody-producing B cells were 2.0 and 2.4 per 10^5^ PBMCs, respectively. Bars indicate the mean, and asterisks indicate statistical significance (*P*<0.05).

Circulating B cells producing IgG anti-GPIIb/IIIa and anti-GPIb antibodies were simultaneously measured in patients with primary ITP, various thrombocytopenic conditions, and healthy controls (Figure 1). No clear spot was detected in the control plates coated with bovine serum albumin alone. There were significantly more circulating anti-GPIIb/IIIa antibody-producing B cells in patients with primary ITP, SLE, liver cirrhosis, and post-HSCT than in healthy controls (5.4±4.7, 6.0±6.4, 10.0±5.8, and 6.3±8.3 versus 0.3±0.4; *P*<0.05 for all comparisons). In contrast, there was no difference in anti-GPIIb/IIIa antibody-producing B cells between the non-ITP disease controls, including aplastic anemia and MDS, and healthy controls. Among ITP-related conditions, patients with liver cirrhosis had a greater frequency of anti-GPIIb/IIIa antibody-producing B cells than did those with primary ITP, SLE, or post-HSCT (*P*<0.05 for all comparisons). Similarly, there were significantly more anti-GPIb antibody-producing B cells in patients with primary ITP, SLE, liver cirrhosis, and post-HSCT than in healthy controls (3.0±3.3, 10.5±25.6, 4.8±5.2, and 3.4±6.2 versus 0.4±0.4; *P*<0.01 for all comparisons). Again, there was no difference between the non-ITP disease controls and healthy controls. The frequency of anti-GPIb antibody-producing B cells tended to be higher in SLE patients than in those with other ITP-related conditions, but the difference was not statistically significant.

**Figure 2:**
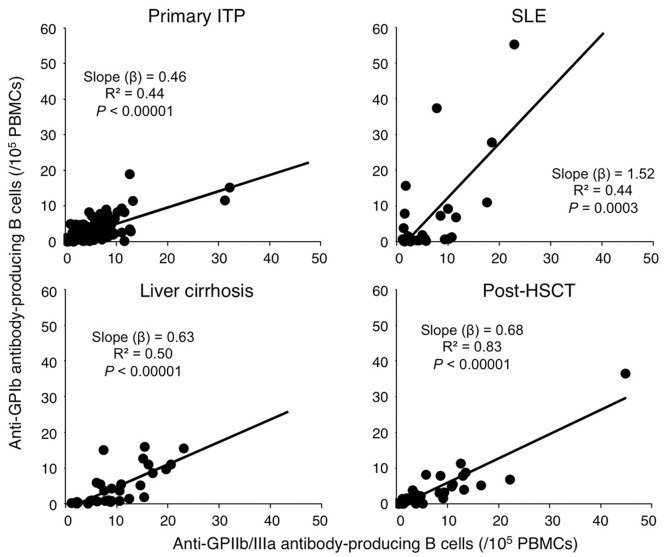
Correlations between circulating anti-GPIb and anti-GPIIb/IIIa antibody-producing B cells in patients with primary ITP, SLE, liver cirrhosis, and post-HSCT.

The circulating frequencies of anti-GPIb and anti-GPIIb/IIIa antibody-producing B cells were correlated with each other in patients with primary ITP, SLE, liver cirrhosis, and post-HSCT (*P*<0.0003 for all correlations) (Figure 2). Based on the slopes of the fitted lines obtained by the single regression model, the anti-GPIIb/IIIa antibody-producing cells exceeded the anti-GPIb antibody-producing B cells in patients with primary ITP, liver cirrhosis, and post-HSCT (slope <1), whereas the anti-GPIb antibody-producing B cells predominated in SLE patients (slope >1).

### Diagnostic Utility of Anti-GPIIb/IIIa and Anti-GPIb Antibody-producing B Cells

To evaluate the diagnostic utility of the anti-GPIIb/IIIa and anti-GPIb ELISPOT assays, the results of these tests were judged as positive or negative based on being above or below a defined cut-off level. We used 2.0 per 10^5^ PBMCs as the cut-off value for anti-GPIIb/IIIa antibody-producing B cells, which was determined in our previous study [7] and 2.4 per 10^5^ PBMCs for circulating anti-GPIb antibody-producing B cells, which was 5 standard deviations above the mean value obtained from healthy controls. The positive frequencies of circulating anti-GPIIb/IIIa and anti-GPIb antibody-producing B cells, and their combination in patients with primary ITP, various thrombocytopenic conditions, and healthy controls are summarized in Table 2. Anti-GPIIb/IIIa antibody-producing cells were detected in 86% of the patients with primary ITP, and in 76%, 97%, and 62% of the patients with SLE, liver cirrhosis, and post-HSCT, respectively. In contrast, the percentages of patients with a positive frequency of anti-GPIb antibody-producing B cells were lower (38–50%) than those of anti-GPIIb/IIIa antibody-producing cells. These antibody-producing cells were infrequently detected in patients with aplastic anemia or MDS. Of 16 patients with primary ITP who were negative for the anti-GPIIb/IIIa antibody-producing cells, 5 (31%) were positive for the anti-GPIb antibody-producing cells. Three (50%) out of 6 SLE patients with the negative anti-GPIIb/IIIa ELISPOT result were positive for the anti-GPIb ELISPOT assay, but none of the patients with liver cirrhosis or post-HSCT who showed the negative anti-GPIIb/IIIa ELISPOT result were positive for the anti-GPIb ELISPOT assay. When the results for anti-GPIIb/IIIa and anti-GPIb antibody-producing B cells were combined, the positive frequency was slightly increased in patients with primary ITP and SLE, but not in those with liver cirrhosis and post-HSCT, because in the latter cases the anti-GPIb antibody-producing B cells always coexisted with anti-GPIIb/IIIa antibody-producing ones.

**Table 2:** Positive frequencies of circulating anti-GPIIb/IIIa and anti-GPIb antibody-producing B cells, and their combination in patients with primary ITP, various thrombocytopenic conditions, and healthy controls.

	Primary ITP(n = 114)	SLE (n = 25)	Liver cirrhosis (n = 30)	Post-HSCT(n = 39)	Aplastic anemia/MDS (n = 18)	Healthy controls (n = 32)
Anti-GPIIb/IIIa antibody-producing B cells alone	86%	76%	97%	62%	17%	0%
Anti-GPIb antibody-producing B cells alone	43%	40%	50%	38%	11%	0%
Anti-GPIIb/IIIa antibody-producing B cells ANDanti-GPIb antibody-producing B cells	38%	28%	50%	38%	11%	0%
Anti-GPIIb/IIIa antibody-producing B cells ORanti-GPIb antibody-producing B cells	90%	88%	97%	62%	17%	0%

SLE, liver cirrhosis, and post-HSCT are conditions potentially causing secondary ITP, whereas aplastic anemia and MDS are non-ITP disease controls.

We then focused on the utility of anti-GPIIb/IIIa and anti-GPIb antibody-producing B cell measurement for the diagnosis of primary ITP. This analysis included 114 patients with primary ITP and 18 with non-ITP thrombocytopenia, including aplastic anemia and MDS. The anti-GPIIb/IIIa antibody-producing B cell measurement had a sensitivity of 86%, specificity of 83%, positive predictive value of 98%, and negative predictive value of 50%. In contrast, the sensitivity of the anti-GPIb antibody-producing cells was only 53%, while the specificity was 89% and positive and negative predictive values were 86% and 20%, respectively. When the two tests were combined, the sensitivity was slightly improved to 90% without effectively reducing the specificity or positive predictive value of the anti-GPIIb/IIIa ELISPOT assay alone (83%, 97%, respectively). When the same analysis was performed in patients with SLE, the sensitivity was improved from 76% in case of the anti-GPIIb/IIIa ELISPOT assay alone to 88% in case of combining the two tests.

### Clinical Characteristics Associated with Anti-GPIIb/IIIa and Anti-GPIb Antibody-producing B Cells in Patients with Primary ITP

Patients with primary ITP were stratified into two groups based on the presence or absence of anti-GPIIb/IIIa or anti-GPIb antibody-producing B cells (Table 3). Newly diagnosed ITP was less common in patients with anti-GPIIb/IIIa antibody-producing B cells, than in those without. The positive anti-GPIb ELISPOT assay result was associated with a low platelet count, lack of *H. pylori* infection, and positive ANA, independent of the cut-off levels. Therapeutic responses to *H. pylori* eradication, IVIG, and splenectomy tended to be worse in patients with a positive anti-GPIb ELISPOT assay than in those without, but only the difference in the response to IVIG reached statistical significance. On the other hand, there were no differences in clinical characteristics except the ITP classification between patients with and without anti-GPIIb/IIIa antibody-producing B cells.

**Table 3:** Clinical findings in patients with primary ITP, stratified by the presence or absence of circulating anti-GPIb or anti-GPIIb/IIIa antibody-producing B cells.

	Anti-GPIIb/IIIa antibody-producing B cells			Anti-GPIb antibody-producing B cells		
	Present (n = 98)	Absent (n = 16)	*P*	Present (n = 49)	Absent (n = 65)	*P*
Sex (% female)	58%	69%	0.60	65%	55%	0.28
Age at examination (years)	50.3±17.5	45.4±14.1	0.29	50.0±17.6	49.3±16.9	0.85
Newly diagnosed ITP (%)	37%	75%	0.009	47%	38%	0.47
Platelet count (x 10^9^/L)	27.5±11.5	31.7±12.1	0.19	19.8±9.4	34.4±8.8	<0.0001
*H. pylori* infection	28%	19%	0.66	14%	35%	0.01
Positive ANA (≥1∶40)	26%	44%	0.23	51%	23%	0.002
Positive ANA (≥1∶160)	17%	13%	0.83	24%	5%	0.004
Therapeutic response						
* H. pylori* eradication	62% (n = 26)	100% (n = 3)	0.50	33% (n = 6)	74% (n = 23)	0.16
IVIG	65% (n = 40)	56% (n = 9)	0.60	46% (n = 24)	80% (n = 25)	0.03
Corticosteroids	21% (n = 53)	17% (n = 12)	0.91	24% (n = 33)	15% (n = 33)	0.54
Splenectomy	76% (n = 37)	75% (n = 8)	0.97	64% (n = 22)	86% (n = 23)	0.14

## Discussion

In this study, we successfully developed an ELISPOT assay for detecting anti-GPIb antibody-secreting B cells, by applying the principles of our previously developed anti-GPIIb/IIIa antibody-producing B cell measurement. Anti-GPIb antibody-secreting B cells were detected in the circulation of patients with primary ITP as well as conditions that potentially cause secondary ITP, but were infrequently found in patients with aplastic anemia or MDS. Thus, the anti-GPIb ELISPOT assay is useful for identifying patients with ITP, but its sensitivity was much inferior to that of the anti-GPIIb/IIIa ELISPOT assay, indicating that detection of anti-GPIb antibody-producing cells could not replace the anti-GPIIb/IIIa assessment in ITP diagnosis. In addition, concomitant measurement of anti-GPIb and anti-GPIIb/IIIa antibody-producing B cells had limited utility: a slight increase in sensitivity only for primary ITP and SLE. These findings indicate that, rather disappointingly, the additional measurement of anti-GPIb antibody-producing B cells on top of the anti-GPIIb/IIIa ELISPOT assay does not improve the diagnostic accuracy for patients suspected of having ITP. However, it may be worth measuring the anti-GPIb ELISPOT assay in patients who are suspected to have primary ITP or secondary ITP in association with SLE, but are negative for anti-GPIIb/IIIa antibody-producing cells, because there is a >30% chance for obtaining the positive result.

Several laboratories have reported antigen-specific assays, such as the monoclonal antibody-specific immobilization of platelet antigen (MAIPA) assay, for detecting autoantibodies to GPIIb/IIIa and GPIb, which are either bound to platelet surfaces or present in plasma, although an international study comparing these antigen-specific assays revealed that good inter-laboratory agreement was obtained only when the platelet-associated antibodies were measured [21]. In this regard, Warner and colleagues reported that an antigen-specific assay detecting platelet-associated anti-GPIIb/IIIa antibodies had a sensitivity of 57% and a specificity of 96% for the diagnosis of primary ITP, and the additional measurement of anti-GPIb antibodies increased the diagnostic sensitivity to 66%, and retained a specificity of 92% [3]. In another report using a prospective cohort of thrombocytopenic patients, platelet-associated anti-GPIIb/IIIa and anti-GPIb antibodies detected by direct MAIPA were present in 49% of 93 patients with ITP, including 74 with the primary form, and in only 22% of 54 patients with non-ITP thrombocytopenia [4]. The platelet-associated antibodies to GPIIb/IIIa (88%) were more frequently directed than to GPIb (52%), while 40% of patients had concomitant antibodies to both of these platelet glycoproteins. Finally, McMillan et al examined platelet-associated anti-GPIIb/IIIa and anti-GPIb antibodies in 282 patients with primary ITP, and found that the majority of patient samples contained platelet-associated antibodies recognizing GPIIb/IIIa alone (52%); fewer reacted to GPIb alone (12%) or to both complexes (15%) [5]. Our findings, obtained by measuring the anti-GPIIb/IIIa and anti-GPIb antibody-producing circulating B cells, were generally concordant with these results from assays detecting specific platelet-associated antibodies: GPIIb/IIIa antibodies were predominantly recognized, while anti-GPIb antibody measurement contributed minimally to the diagnosis of primary ITP. The ELISPOT assays appear to be more sensitive than the platelet-associated antigen-specific assays (90% versus 49–66%), but prospective studies comparing the ELISPOT assays with other anti-platelet autoantibody detection tests are necessary to confirm this.

The main reason for the low utility of anti-GPIb antibody-producing B cells for the diagnosis of ITP is that circulating anti-GPIIb/IIIa and anti-GPIb antibody-producing B cells coexist in the majority of patients with ITP. Only a small number of patients had anti-GPIb antibody-producing B cells alone. Therefore, in routine clinical settings, the anti-GPIIb/IIIa ELISPOT assay appears to be sufficient for the diagnosis of ITP. Interestingly, the levels of anti-GPIb and anti-GPIIb/IIIa antibody-producing B cells in the circulation were correlated with each other in ITP patients, irrespective of whether the diagnosis was primary ITP or one of the secondary forms. These findings indicate that the autoimmune response in the majority of ITP patients targets multiple platelet glycoproteins, which might be a consequence of “epitope spreading” [2]. In this regard, we have proposed a “pathogenic loop” model for the ongoing anti-platelet autoantibody response in ITP patients [22]. Namely, macrophages in the reticuloendothelial system (spleen in the majority of the patients) capture opsonized platelets, and activate autoreactive T helper cells that stimulate the B cells to proliferate [23], differentiate into plasma cells [24], and produce anti-platelet autoantibodies, which in turn bind to circulating platelets. The continuous destruction of platelets in the reticuloendothelial system would allow the processing and presentation of a whole panel of platelet antigens by macrophages, some of which could elicit additional autoreactive T cell responses, resulting in the production of autoantibodies against other platelet glycoproteins.

In primary ITP and various forms of secondary ITP, SLE was unique in having a predominant anti-GPIb antibody-producing B cell response. SLE is a systemic autoimmune disease characterized by a loss of tolerance to nuclear and other self-antigens, a production of pathogenic autoantibodies, and damage to multiple organ systems [25]. Taken together with the association between anti-GPIb antibody-producing B cells and the production of ANAs, even in patients with primary ITP, the anti-GPIb autoantibody response might be linked to systemic autoimmunity. Because a significant proportion of SLE patients had anti-GPIb antibody-producing B cells in the absence of anti-GPIIb/IIIa antibody-producing B cells, measurement of the anti-GPIb in addition to anti-GPIIb/IIIa antibody-producing B cells may have some merit for accurately identifying secondary ITP in patients with SLE and thrombocytopenia, although the number of patients analyzed in this study was too small to draw a firm conclusion.

Much effort has been made to identify clinical associations of individual anti-platelet glycoprotein antibodies, but the clinical significance of such antibodies remains uncertain. In patients with primary ITP, the presence of platelet-associated anti-GPIb antibodies was shown to be associated with a lower platelet count [26], [27] and inadequate responses to corticosteroids [26] and IVIG [28]. Our results were consistent with these previous observations, including the low platelet count and poor responses to therapeutic interventions, especially to IVIG. In this regard, some monoclonal antibodies against GPIb are known to induce platelet activation, which may lead to accelerated platelet destruction independent of the Fcγ receptor-mediated process in ITP patients [29]. We additionally found correlations between anti-GPIb antibody-producing B cells and a low prevalence of *H. pylori* infection or a high frequency of positive ANA. These findings indicate that there may be a relatively homogeneous subset of primary ITP cases defined by the anti-GPIb antibody response, and characterized by severe thrombocytopenia, the absence of *H. pylori* infection, a positive ANA, and a poor therapeutic response.

In summary, our ELISPOT assay for detecting anti-GPIb antibody-secreting B cells is useful for identifying patients with ITP, but its utility for diagnosing ITP is apparently inferior to the anti-GPIIb/IIIa ELISPOT assay. Nevertheless, detection of the anti-GPIb antibody response is useful for subtyping patients with primary ITP and predicting the therapeutic response.
